# Care for Amish and Mennonite children with cystic fibrosis: a case series

**DOI:** 10.1186/1471-2431-9-4

**Published:** 2009-01-15

**Authors:** Jonathan F Henderson, Ran D Anbar

**Affiliations:** 1 Department of Pediatrics, State University of New York Upstate Medical University, Syracuse, NY, USA

## Abstract

**Background:**

Published articles have described a lack of willingness to allow preventative measures, as well as other types of modern therapies, as an obstacle to providing medical care for Amish and Mennonite populations.

**Methods:**

We present data regarding the 12 Amish and Mennonite patients at the SUNY Upstate Medical University Pediatric Cystic Fibrosis Center and three representative case reports.

**Results:**

Families of patients from these communities receiving care at our Center have accepted preventive therapy, acute medical interventions including home intravenous antibiotic administration, and some immunizations for their children with cystic fibrosis, which have improved the health of our patients. Some have even participated in clinical research trials. Health care education for both the child and family is warranted and extensive. Significant Cystic Fibrosis Center personnel time and fundraising are needed in order to address medical bills incurred by uninsured Amish and Mennonite patients.

**Conclusion:**

Amish and Mennonite families seeking care for cystic fibrosis may choose to utilize modern medical therapies for their children, with resultant significant improvement in outcome.

## Background

While the Amish and Mennonite communities offer the opportunity for study of closed populations with an increased incidence of certain genetic disease and defects, including cystic fibrosis (CF) [1-4], no published data have dealt with treatment and socioeconomic impact of CF on Amish and Mennonite families.

In the United States, the population of the Amish in 2001 was reported as approximately 200,000 while the Mennonite population was approximately 250,000 [5]. The incidence of CF among these populations has been difficult to estimate because of the closed nature of their communities. For example, in one Ohio Amish isolate the incidence of CF was 1/569 live births, while in another isolate there was no occurrence of CF among 4448 live births [6]. The incidence of CF in the general United States population has been approximately 1/3500 live births, however this incidence is falling, perhaps as a result of preconception and prenatal screening offered to the general population in order to identify carriers of cystic fibrosis [7]. As a majority of Amish and Mennonites may approve of CF carrier testing that can impact whether CF carriers marry each other [1], it is possible that these populations also are experiencing a decline in the incidence of CF. However, the extent of genetic testing and counseling services available to these communities is unclear [8].

The Mennonites separated from the "Old Order" Amish in 1850 as a result of adoption of new practices by the Mennonites, however, many of the central tenants of each group remain similar [9]. Both the Amish and Mennonites believe that good health is a gift from God, resulting from hard work and strict obedience to the teachings of the Bible. The ability to work defines a "healthy" individual [9-12]. Conversely, illness is generally believed to be "God's will," while death is considered a natural part of life and a new beginning, rather than an end or punishment [12]. The Amish and Mennonites believe in the strict separation of church and state, which historically has extended to refraining from use of government funds, including Medicaid and social security [11,12]. Further, typically they do not purchase commercial insurance [12]. Thus, barriers to provision of modern medical care include reimbursement issues, as well as the Amish repudiation of worldly conveniences such as telephones, electricity, and automobiles [12].

In most instances of illness, the Amish rely on folk remedies and herbal medications, among other types of "alternative" care [12]. Patients coming to modern medical facilities typically do so with chronic illness of many years, only after symptoms have become severe and herbal remedies have not proven beneficial [9]. Such willingness has been attributed to a lack of trained professionals within their own communities to deal with severe illness [11,12].

Contrary to the implications of the literature, we present data regarding the 12 Amish and Mennonite patients at the SUNY Upstate Medical University Pediatric CF Center (8% of our CF patients) along with three representative case reports in order to alert physicians that families of these patients can be receptive to modern medical therapy, including preventive measures, which can greatly benefit the patients. Table 1 shows the proportion of our Amish and Mennonite patients who have accepted the recommended standard therapies for CF at our Center. Nine of the patients have undergone genotype testing and found to be homozygous for the ΔF508 CF mutation. Five of the patients have participated in clinical research trials through our Center.

**Table 1:** Rate of standard CF therapy use by 12 Amish and Mennonite patients at our Center

Chest physiotherapy	
Manual percussion	8%

High frequency chest wall oscillator (vest)	86%

Nebulized mucolytic therapy with rhDNase	86%

Multivitamins fortified with vitamins A, D, E, and K	86%

Pancreatic enzymes	100%

Nutritional supplementation	64%

Antibiotic therapy	100%

Due to the small nature of the Amish and Mennonite communities, we were concerned that members of their communities could identify the patients in the case reports. Further, we were concerned by the potential impact of the current article on such communities [13]. Therefore, following consultation with the SUNY Upstate Institutional Review Board (IRB), the families of the patients described in the case reports and their community elders reviewed and approved this manuscript prior to its submission for publication, in order to minimize risk of group harm, and harm to the families involved in each of the three presented cases. Approval of the manuscript also was obtained from the SUNY Upstate IRB.

## Case Reports

### Patient A – Amish

Patient A was four-years-old when he began receiving care at our Center. He had multiple siblings, including an older, much healthier sister who had CF. At the start of therapy, the patient's parents considered the long-term prognosis of their son when deliberating what treatments should be used. Would the disease be painful? What help, if any, would medications and other medical technology provide that herbal therapies had not? Is treatment futile in children with CF?

Following lengthy conversations involving not only the patient's parents, but also the bishop and elders in his community, it was felt to be in Patient A's best interest to begin care with standard therapy for CF at our Center (Table 1). A gas-powered generator was used to power the vest. Patient A, as well as all of our Amish and Mennonite patients, qualified for pharmaceutical companies' patient assistance programs for many of his medications. Those medications not covered under individual patient assistance programs were secured from pharmaceutical company representatives in the form of samples. Our hospital established a fee reduction program to help offset the cost of outpatient visits and inpatient hospital admissions for these families, all of whom qualified based on their income level. Notably, all of the aforementioned assistance was secured by our CF Center social worker, who was vital in communicating with the parents and helping them complete required paperwork.

While standard therapies appeared to slow progression of the patient's lung disease, when his status worsened it was recommended by our Center physician that intravenous antibiotic therapy be instituted. The family consulted with their elders who recommended that such therapy be withheld because of its cost, and as use of such therapy would only prolong the dying process in a patient with a terminal disease. During discussions with the family, our Center physician stated that he believed the patient still would have a reasonable quality of life for several months or even a few years with use of intravenous antibiotics. Further, withholding of antibiotic therapy at the time of the discussions would result in a much longer dying process than if the patient lived a longer life with concomitant lung disease progression prior to withholding of aggressive therapy. After a number of discussions at our Center, the patient's parents allowed the introduction of intravenous therapy. They stated that if the medications did not seem to help their son's symptoms, it would be "God's will." At such a point they would not want to give him further intravenous therapy. Home intravenous therapy was instituted because the family could not afford to pay in-patient hospital charges.

Several courses of intravenous antibiotic therapy were associated with a significant improvement of the patient's respiratory condition. However, after two years, he failed to respond to two intravenous antibiotic courses. At that time, the decision was made to withhold further intravenous therapy. The patient was provided supplemental oxygen at home that was generated by a concentrator, as well as on-going vest therapy. At the invitation of the patient and his family, a physician, respiratory therapist, and social worker from our Center made home visits to check on the patient as he deteriorated.

As he grew sicker, the patient was prescribed oral narcotics to be used as necessary for discomfort. Four months after withholding intravenous therapy, following eight years of treatment at our Center, Patient A died. According to his family, as has become custom within this community, all of his supplies, including the vest and medicines, were given to another child in the community with CF; in this case, his sister. The strong bond between the family and providers at our Center has led to Amish from other communities in New York to seek care at our Center at this family's advice.

### Patient B – Mennonite

At the age of two months, Patient B was seen at another Center for failure to thrive, emesis, bloody stools, rash and bruising as a result of a vitamin K deficiency. Following an episode of significant cough and wheezing, he was diagnosed with CF. Later, it was found that Patient B also had biliary cirrhosis.

After receiving care elsewhere for four years, the patient came to our Center because his family became discontented with the medical care they had been receiving. The parents stated they were unhappy because they were not provided with accurate information regarding the patient's health condition, need for testing, and treatment options. Reportedly, the family was told that the patient would require hospitalization every few months for his entire life, as well as a liver transplant. The parents reportedly felt much pressure from staff members of the other institution to obtain State Aid because of the high on-going and projected costs of his medical care. Moreover, herbal remedies that patient B had used since he first became ill were discouraged by his physician, even though the family felt his caregivers did little to understand the need for these remedies.

After transferring to our Center, the parents of patient B were willing to try therapies they had heard worked well in others with CF in their community, including standard therapies offered at our Center (Table 1). According to these parents, their readiness to try such therapies, including those that they may have previously rejected, was the result of the willingness of our Center staff to discuss the potential benefit and harm of standard as well as alternative therapies. For example, our CF Center physician was open to use of herbal therapies for patient B, although he informed the family that no studies have demonstrated the effectiveness of treatment of CF. Notably, the deacon and some of the bishops of patient B's Mennonite community actively discouraged use of the herbs because their use was thought to represent witchcraft. This created significant discomfort between the family and their bishop. The parents felt that while some of their community were supportive of herbs and would be willing to help fund this therapy, they felt uncomfortable asking for financial resources from other community members. Ultimately, without pressure from our Center, the family decided to obtain insurance coverage through Medicaid.

Four months after institution of our standard CF therapies and nebulized tobramycin (TOBI^®^) in treatment of the patient's airway colonization with *Pseudomonas aeruginosa*, the patient's pulmonary crackles cleared, and his hemoglobin saturation in room air rose from 93% to 96%. His body mass index over the same time interval increased from15.1 kg/m^2 ^(25^th ^percentile for age) to 16.2 kg/m^2 ^(75^th ^percentile for age).

Helping others within their community with CF was an important aspect of Patient B's overall care, according to his parents. Therefore, they consented to his inclusion in the CF Foundation national patient registry, which tracks demographics of CF patients throughout the United States. Moreover, because of the significant history of biliary disease in their child, the parents also consented to his enrollment in a gene modifier study of patients with CF liver disease, for which participation consisted of provision of a single blood specimen.

### Patient C – Amish

Patient C was diagnosed with CF following postnatal testing done as a result of a sibling with the disease. She showed few symptoms of CF until she was two-years-old, at which time she was brought by her parents to a hospital in Michigan because she was "pale". While she did not have any significant respiratory disease prior to this admission, her condition warranted the start of treatment for CF including rhDNase, and a bronchodilator, which largely was covered under Michigan's Trust Fund for Children with Special Needs (formerly the Crippled Children's fund).

Shortly thereafter, the patient and her family relocated to another state, where coverage for her expensive therapies was less available. More importantly to the family, they "didn't like the doctor" taking care of their children in the new CF Center because they felt he did little to understand the beliefs of their culture, and why certain therapies are accepted while others are not. After talking to others in their community, the parents of Patient C were told to come to our Center because of the work we have done with the Amish. A generator was installed by the family for the use of a nebulizer and vest. Medications needing refrigeration have been kept on ice.

Three years ago, the family was contacted by an individual who sought to, "help Amish children with CF." The family was presented with a plan to help cover the costs of treatment and were told that it "did not involve government assistance." Ultimately, this plan turned out to be Medicaid through the state of New York, which caused distress for this family, when they learned about this at a subsequent visit to our Center. The social worker at our Center worked to amend this problem with the State, and assured the elders and others within their community that the family in fact did not knowingly apply for state or governmental aid. The family of Patient C was advised by the social worker to refrain from applying for any sort of "help" from others outside our facility without first contacting our Center.

The parents consented to have Patient C participate in two studies, including one involving growth hormone. The latter study provided reimbursement for the family's transportation costs to our Center and there were no charges associated with the Center visits for the duration of the study. The family also agreed to allow immunization for influenza virus on a yearly basis. This family explained that even though Amish patients often have refused immunizations of any kind, such preventative care currently is being left to the family's discretion as attitudes towards preventative care among the Amish have shifted significantly over the past decade.

## Discussion

Publications have described a lack of willingness to allow preventative measures as an obstacle to providing modern medical care for Amish and Mennonite populations [10-12]. Based on the experiences reported by our patients, this information may have led some within the medical community to assume that Amish and Mennonite families are unwilling to allow preventative care, as well as other types of modern therapies, which has resulted in provision of suboptimal care for patients with CF.

Nonetheless, it is evident that Amish and Mennonite families can be open to effective, modern therapy for this disease. After extensive exploration of the beliefs and expectations of families from these communities, they have accepted preventive therapy, acute medical interventions including home intravenous antibiotic administration, and some immunizations for their children with CF. Some even have participated in clinical research trials.

Significant CF Center personnel time and fundraising are needed in order to address medical bills incurred by uninsured Amish and Mennonite patients with chronic disease such as CF. Several options are available. Churches within the community often have fundraisers, through selling Amish and Mennonite foods, quilts and furniture. "Amish Aid" is a type of medical insurance governed by businessmen within the Amish church to help pay for hospital bills [3]. Such funds are used by members of the church community, but often fall short of covering costs. With the availability of programs such as our institution's fee reduction program to offset costs, caring for Amish patients becomes more manageable, and helps these families to seek care.

Health care education for both the child and family is warranted and extensive. By openly discussing the rationale for state-of-the-art and alternative treatments, side effects and outcomes, Amish and Mennonite families can embrace state-of-the-art medical therapies, with significant positive results. Participation of Amish and Mennonite patients in some of our clinical research trials also has been very helpful in that participating patients are reimbursed for their transportation costs, and clinic charges are minimized for the duration of studies sponsored by pharmaceutical companies. Further, participation in such trials allows for more frequent visits at the CF Center for the enrolled patients as well as siblings with CF, which leads to provision of more timely and thus improved health care.

As presented in this report, some members of each group also are willing to allow certain preventative measures. A recent study by Yoder and Dworkin [14], in which questionnaires were mailed to all households in an Illinois Amish community, showed that the majority of the community vaccinates all (84%) or some (12%) of their children, with only a small minority objecting due to concerns about vaccine safety, and an even smaller cohort objecting due to religious reasons. Thus, Amish families often are willing to utilize vaccination as a form of preventative care.

This report illustrates that health care geared to the cultural needs of patients and their families can lead to an improved outcome. It has been proposed that cultural sensitivity improves establishment of rapport and thus promotes cooperation and adherence to therapy [15]. Such sensitivity should include assessment of the patients' preferences and beliefs, and adjustment of health care delivery accordingly [16]. For example, in some cultures more emphasis is placed on collective rather than individual decision making, communication patterns may differ (e.g., there may be a relative emphasis on non-verbal communication), and views may differ regarding physicians, suffering, and the afterlife [16].

## Conclusion

It appears essential for the health care team to understand, consider, and incorporate current beliefs of Amish and Mennonite communities into the development of effective programs for treatment of their members with CF. Similar efforts should be undertaken whenever health care providers encounter patients from different cultures or religions.

## Abbreviations

CF: cystic fibrosis

## Competing interests

The authors declare that they have no competing interests.

## Authors' contributions

JH wrote the manuscript, and it was edited by RA, who was the attending physician for the described patients. Both of us approve the submission of this version of the manuscript, and take full responsibility for it.

## Pre-publication history

The pre-publication history for this paper can be accessed here:


